# Sub-Second Temporal Integration of Vibro-Tactile Stimuli: Intervals between Adjacent, Weak, and Within-Channel Stimuli Are Underestimated

**DOI:** 10.3389/fpsyg.2017.01295

**Published:** 2017-07-31

**Authors:** Scinob Kuroki, Takumi Yokosaka, Junji Watanabe

**Affiliations:** NTT Communication Science Laboratories, Nippon Telegraph and Telephone Corporation Kanagawa, Japan

**Keywords:** time perception, temporal judgment, grouping, somatotopy, vibro-tactile

## Abstract

Tactile estimation of sub-second time is essential for correct recognition of sensory inputs and dexterous manipulation of objects. Despite our intuitive understanding that time is robustly estimated in any situation, tactile sub-second time is altered by, for example, body movement, similar to how visual time is modulated by eye movement. The effects of simpler factors, such as stimulus location, intensity, and frequency, have also been reported in temporal tasks in other modalities, but their effects on tactile sub-second interval estimation remain obscure. Here, we were interested in whether a perceived short interval presented by tactile stimuli is altered only by changing stimulus features. The perceived interval between a pair of stimuli presented on the same finger apparently became short relative to that on different fingers; that of a weak-intensity pair relative to that of a pair with stronger intensity was decreased; and that of a pair with the same frequency relative to one with different frequencies was underestimated. These findings can be ascribed to errors in encoding temporal relationships: nearby-space/weak-intensity/similar-frequency stimuli presented within a short time difference are likely to be integrated into a single event and lead to relative time compression.

## Introduction

Accurate estimation of sub-second time is a critical challenge for the brain. Vision research on perception of this time range has repeatedly demonstrated plasticity and a lack of robustness by introducing temporal illusions (Nishida and Johnston, 2002; Morrone et al., 2005; Ibbotson et al., 2006, 2007; Eagleman, 2008; Terao et al., 2008). The apparent timing of visual events is not robust with manipulations of low-level stimulus features (Kanai et al., 2006; Xuan et al., 2007; Terao et al., 2008), suggesting that our visual time perception depends on how the brain groups/binds multiple inputs as single or multiple events. In touch, on the other hand, few studies have investigated this issue despite the fact that tactile temporal estimation of this range is also essential for accurate object recognition and the rapid execution of sensorimotor actions.

For tactile brief-interval estimation, body movement can alter the perceived interval (Tomassini et al., 2014). When a pair of tactile stimuli is presented just before and during movement, the apparent interval between them is reduced, which is in line with the modulation of the perceived timing of visual events by eye/body movement (Yarrow et al., 2001; Haggard et al., 2002; Yarrow and Rothwell, 2003; Hagura et al., 2012; Yokosaka et al., 2015). However, the effects of simpler stimulus attributes on tactile sub-second interval estimation, such as the location of the stimulation and the stimulus intensity and frequency, have not been investigated. Since grouping/binding is based on the similarity of stimuli and their spatial and/or temporal proximity (Festinger et al., 1970), it may also be worthwhile to consider the role these parameters play in brief-interval estimation. Here, we examined whether such stimulus attributes contribute to the binding of multiple tactile inputs, and if so, to what extent they affect perceived brief time intervals in touch.

Our first concern was the effect of stimulus location, in particular, the inter-stimulus distance, on brief-interval estimation. Tactile input is encoded by peripheral mechanoreceptors, which are distributed over the entire surface of the body. Thus, it takes inputs at different locations on the skin different amounts of time to reach the brain. Previous studies revealed that a single input at different locations induces different perceived timings (von Bekesy, 1959; Gilson, 1969; Geffen et al., 2000; Harrar and Harris, 2005; Kuroki et al., 2010), and, importantly, that the difference in the perceived timing does not match the latency difference of each stimulus. Thus, the effect of stimulus location on tactile temporal judgment cannot be simply ascribed to differences in neural transmission time. For multiple inputs, the accuracy of simultaneity judgments improves when the stimuli are presented on a single finger compared to when they are presented on two fingers (Clark and Geffen, 1990; Kuroki et al., 2010), and temporal order judgments become accurate with spatial separation of the stimuli (Craig and Baihua, 1990; Shore et al., 2005; Kuroki et al., 2010). In addition, the occurrence probability and perceived intensity of apparent motion differ according to the stimulus location (Sherrick and Rogers, 1966; Sherrick, 1968; Kirman, 1974; Kuroki et al., 2010). These previous findings are in line with the speculation that there is less binding of stimuli when they are separated in the spatial domain (Festinger et al., 1970). Here, considering the estimation of the temporal interval between two stimuli, separating them may result in apparent overestimation. To test for this, we compared the perceived interval between a pair of tactile stimuli presented to the same finger and to different (next) fingers (experiment 1). We measured participants’ performance for pairs of 30-Hz vibrations (low-frequency condition) and pairs of 300-Hz vibrations (high-frequency condition). The vibro-tactile system encodes mechanical input in a frequency-dependent manner with mechanoreceptor-afferent channels. The rapidly adapting (RA) afferent channel is sensitive to lower vibration frequencies (peaks around 30 Hz), and the Pacinian-corpuscle (PC) channel is sensitive to higher frequencies (peaks around 250 Hz) (Talbot et al., 1968; Mountcastle et al., 1972; Freeman and Johnson, 1982; but see also Johansson et al., 1982). Not only the responding mechanoreceptors but also the impression of sensation differs according to the frequency range: low-frequency vibration induces a “flutter” sensation, while high-frequency vibration induces a “vibration” sensation (Werner and Mountcastle, 1965; Talbot et al., 1968 ; Mountcastle et al., 1990). The characteristics of temporal/spatial summation also differ according to the frequency range: the detection threshold decreases as stimulus duration/size increases only with high-frequency vibration (Verrillo, 1965; Gescheider, 1976). Thus, we were interested in whether the difference in frequency range differently affects the location effect on tactile brief-interval estimation.

Our second concern was the effect of intensity. One visual study has shown that reducing the visibility of presented stimuli (two flashes) could cause apparent underestimation of their temporal interval (Terao et al., 2008). Although the latencies of the two stimuli do not change, the visual sensory system is likely to integrate stimuli with low visibility as one. A similar trend has been observed in auditory studies (Gescheider, 1966; Divenyi and Danner, 1977; Matthews et al., 2011). In touch, it has been suggested that intensity suppression and temporal interval compression are not independent (Juravle, 2015), but this hypothesis still awaits further validation. We hypothesized that, as with visual stimuli, reducing intensity results in stronger binding of tactile stimuli and thus in apparent underestimation of the temporal interval of the two vibrations. To test this, we adopted a paradigm similar to that in visual studies: Participants compared the perceived intervals, one of whose amplitude was half as large as the other (experiment 2). We also measured the performance in the low- and high-frequency conditions.

Finally, we were interested in the effect of frequency differences between paired stimuli on the perceived interval. In the auditory modality, the frequency/pitch of the stimuli and their perceived duration are not perceptually independent (Kryter and Pearsons, 1963; Watson and Gengel, 1969). Further, as mentioned above, subjective impressions of tactile low- and high-frequency stimuli are differently described as flutter and vibration. Thus, it would be more natural for the brain to group two stimuli with the same frequency range rather than two with a different frequency range. To test this possibility, we had participants compare the perceived temporal interval between a pair of low- and high-frequency vibrations and a pair of high-frequency vibrations (experiment 3).

## Materials and Methods

### Participants

One of the authors (SK) and seven volunteers (four men and four women, 29–44 years old, all right-handed) participated in the main experiments. Five volunteers who did not participate in the main experiments and one of the authors (SK; two men and four women, 21–35 years old, all right-handed) participated in a subsidiary experiment. The volunteers had no specialized knowledge about psychophysical experiments and were unaware of the purpose of the experiments. They gave written informed consent before the start of the experiment. Recruitment of participants and experimental procedures were approved by the NTT Communication Science Laboratory Research Ethics Committee and were conducted in accordance with the Declaration of Helsinki.

### Apparatus

**FIGURE 1:**
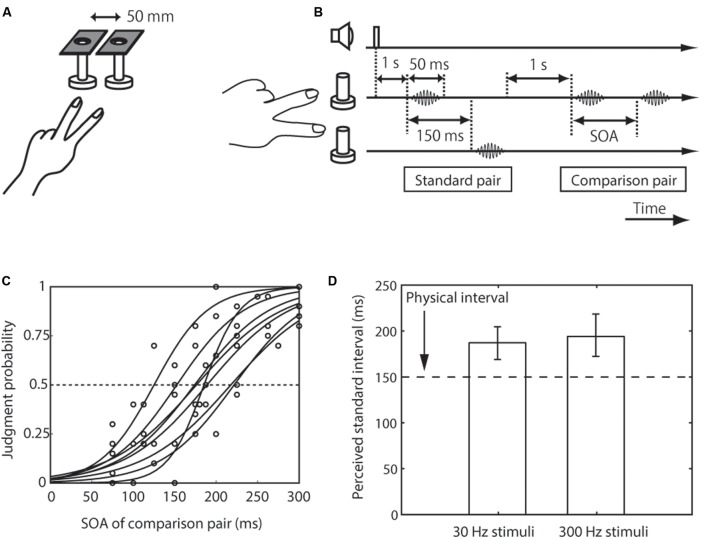
Setup, trial sequence, and results for the inter-stimulus distance effect on tactile temporal perception (experiment 1). **(A)** Schematic representation of the experimental setup. **(B)** Trial sequence. The standard pair was presented on different fingers, and the comparison pair was presented on the index or the middle finger. The frequency of the standard pair and comparison pair was the same. The order of the presentation of the standard pair to the finger (the index finger first or the middle finger first) was randomized. The presentation order of the standard and comparison pairs was randomized. **(C)** Psychometric functions obtained with the 30-Hz stimuli condition. Circles represent the mean of 20 trials for each participant for each SOA. Each line indicates the line fitted with the cumulative Gaussian functions for each participant. **(D)** Averaged PSEs of intervals between the standard pair across the eight participants. The left bar represents trials in which the presented frequency was 30 Hz; and right bar represents trials in which it was 300 Hz. The error bars represent 95% confidence intervals, which were calculated by the bootstrap method (Efron and Tibshirani, 1994).

The apparatus was identical to that in our previous work (Kuroki et al., 2013). Tactile stimuli were delivered to the finger pad with a stack-type piezoelectric actuator (ASB680, NEC Tokin, Japan). The resonance frequency of the actuator was 8 kHz and the maximum load was 800 N. The actuator can accurately produce the necessary displacement with a tolerance of few nanometers. Owing to the actuators large output force and roughly flat frequency response, its movement was accurate irrespective of the force from the participant’s finger within the frequency range we used. The actuator vertically deformed the skin through a hole in a metal board as shown in **Figure 1A**. The diameters of the actuator and the hole were 12.0 and 14.0 mm, respectively. The rigid surround localized the strain energy of the vibration by preventing the spread of skin surface waves (Verrillo et al., 1983). In the distance-effect experiment (experiment 1), a pair of these actuators was used with separate/independent metal boards.

A participant sat at a table with the left arm on a resting box and placed the finger(s) on the actuator(s). The actuator was in contact with the finger throughout the experiment. Participants made responses by clicking a mouse with their right hand. They performed experiments with their eyes open to maintain their arousal level, but they could not see the vibration of the stimulator because their hand and the resting box worked as an occluder. They wore earplugs and headphones, through which white noise was continuously presented throughout the experiment to mask any subtle sound made by the tactile stimulation. Auditory cues were also presented through the headphones.

### Stimuli

We used 30-μm 30-Hz sinusoidal vibrations as low-frequency stimuli and 4-μm 300-Hz vibrations as high-frequency stimuli. The amplitude of low-frequency stimuli was 10 times above the detection threshold (Kuroki et al., 2013), and that of high-frequency stimuli was matched in perceptual intensity based on the results of a preliminary experiment. The duration of the vibration was 50 ms, and its waveform was modulated with a 20-Hz raised cosine window at onset and termination. Thus, the amplitude of vibration gradually rose in the first 25 ms and then gradually decayed in the last 25 ms. In the intensity-effect experiment (experiment 2), the standard pair was a half-amplitude stimuli pair (15 μm for the 30-Hz vibrations and 2 μm for the 300-Hz vibrations).

### Procedure

Participants made a two-alternative forced choice of the longer interval between the two vibrations pairs (**Figure 1B**). At the beginning of each trial, a beep was sounded. The first pair of vibrations was presented 1000 ms after the beep, and the second one was presented after another 1000 ms. As a standard pair, we presented two vibrations with the stimulus onset asynchrony (SOA) of 150 ms. As a comparison pair, we presented two vibrations whose SOA was chosen from seven different levels between 75 and 300 ms on the basis of preliminary experiments performed individually before the main experiment. The presentation order of the standard and comparison pairs was randomized. The participant made a binary response (the interval between the vibrations of the first pair or second pair was longer) about the perceived interval (SOA) between vibrations.

**FIGURE 2:**
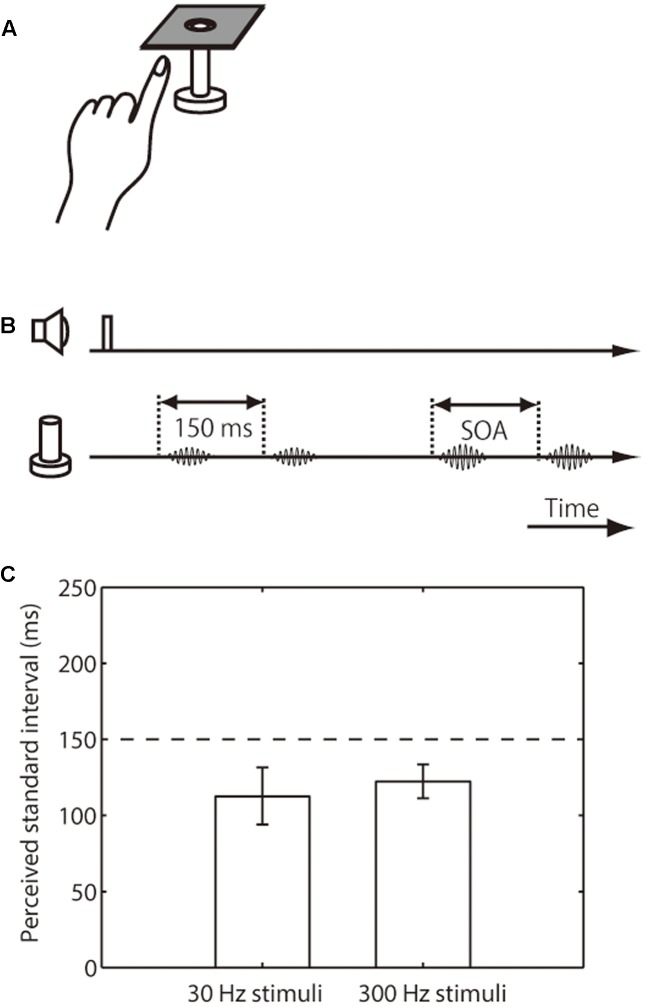
Setup, trial sequence, and results for the intensity effect on tactile temporal perception (experiment 2). **(A)** Schematic representation of the experimental setup. **(B)** Trial sequence. The intensity of the standard pair was half that of the comparison pair. The presentation order of standard and comparison pair was randomized. **(C)** Averaged PSEs of intervals between the standard pair. The left bar represents trials in which the presented frequency was 30 Hz; the right bar represents those in which it was 300 Hz. The error bars represent 95% confidence intervals.

In the distance-effect experiment (experiment 1), each vibration of the standard pair was presented on the index and middle finger of the left hand, while both vibrations of the comparison pair were presented either on the index or the middle finger of the left hand (**Figure 1B**). The finger receiving the first vibration of the standard pair, the one receiving the comparison pair, and the pair presented first were all randomized across trials. The stimulus intensity and frequency of the standard pair and comparison pair were the same and fixed during a block. In the intensity-effect experiment (experiment 2), the intensity of the comparison pair was the same as in experiment 1, but that of the standard pair was halved (**Figure 2B**). The stimulus site of the standard and comparison pairs was the left-hand index finger. The frequency of both pairs was the same and fixed during a block. In the frequency-effect experiment (experiment 3), the standard pair was composed of low- and high-frequency stimuli. Two kinds of standard pairs were tested: A low-high pair composed of a 30-Hz vibration followed by 300-Hz vibration and a high-low pair composed of a 300-Hz vibration followed by 30-Hz vibration. Only one of these standard pairs was presented during a block. The frequency of the comparison pair was fixed at 300 Hz because previous findings suggested that high-frequency stimuli play a dominant role in tactile temporal judgments (Kuroki et al., 2016). The intensity and stimulus site of the standard and comparison pairs were the same as for the comparison pair in experiment 1. Each participant performed 3 effect × 2 base frequency or presented frequency order × 7 SOAs × 20 repetitions. One block consisted of 35 trials, and there were 24 blocks in total.

We conducted a subsidiary experiment, which was identical to the distance-effect experiment (experiment 1) except that an auditory tone pair was used for as the comparison pair. For the standard pair, each vibration was presented on the index and middle finger in the two-finger condition, and both vibrations were presented on the index or middle finger of the left hand in the one-finger condition. The comparison pair comprised two tones (middle C, 50 ms), and the SOA was chosen from seven different levels between 75 and 300 ms. Each participant performed 2 fingers × 2 base frequency × 7 SOAs × 20 repetitions. One block consisted of 35 trials, and there were 16 blocks in total.

### Data Analysis

The rate of the responses in which the interval in the comparison pair was judged to be longer than that in the standard pair was plotted as a function of the SOA of comparison pair. A psychometric function was derived by fitting the cumulative Gaussian function to the data obtained for each condition for each participant using the maximum likelihood method. We determined the SOA that yielded 0.5 in the ordinate as the point of subjective equality (PSE). We calculated 95% confidence intervals (CIs) of PSEs from *N* = 1000 bootstrap estimates (Efron and Tibshirani, 1994) to see whether the perceived interval in the standard pair was over/underestimated compared to that in the comparison pair with 150 ms. In experiment 3 and the subsidiary experiment, we calculated CIs for the difference in the value of the PSE in the two conditions to check whether the PSE differed depending on the conditions.

## Results

### Experiment 1: Inter-Stimulus Distance Effect on Tactile Temporal Perception

We first measured the perceived interval between paired vibrations presented on different fingers (the standard pair, whose interval was fixed at 150 ms) relative to that on the same finger (the comparison pair, whose interval was varied) (**Figure 1A**). Eight participants made a two-alternative forced choice of which interval between the pair of vibrations was longer, the first or the second. The results showed that the apparent interval (i.e., PSE) of the pair presented over different fingers was substantially overestimated compared to the pair on the same finger with the 150-ms interval (i.e., the lower limit of the CI was above 150 ms) (**Figures 1C,D**). This PSE shift was observed when the frequencies of the stimuli were 30 Hz (mean 188 ms, CI [169, 205]) and 300 Hz (mean 195 ms, CI [172, 219]).

### Experiment 2: Intensity Effect on Tactile Temporal Perception

We next examined the perceived interval of the weak-intensity pair relative to that of the base-intensity pair. The amplitude of the weak pair was half that of the base-intensity pair, and both pairs were presented on the index finger of participants’ left hand (**Figures 2A,B**). The results showed that the apparent interval in the weak standard pair was substantially underestimated (i.e., upper limit of CI was below 150 ms), regardless of the stimulus frequency range (mean 113 ms, CI [94, 132] for 30 Hz; mean 123 ms, CI [111, 134] for 300 Hz) (**Figure 2C**).

### Experiment 3: Frequency Difference Effect on Tactile Temporal Perception

**FIGURE 3:**
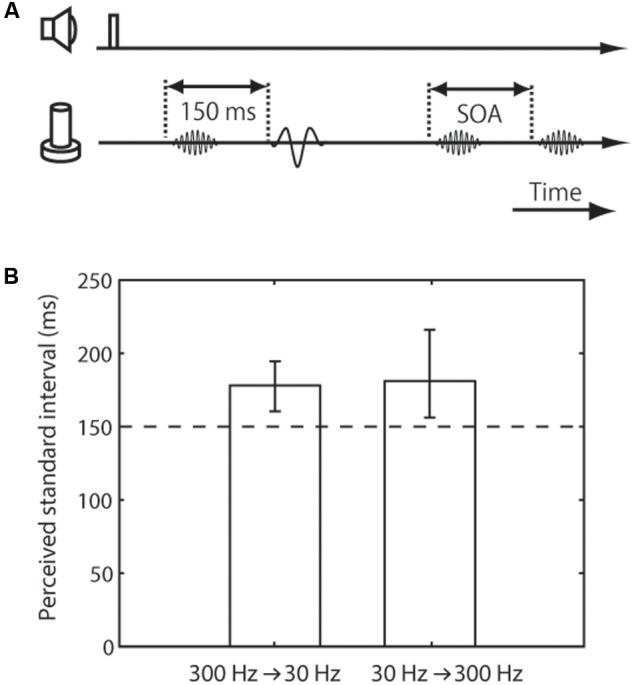
Trial sequence and results for the frequency difference effect on tactile temporal perception (experiment 3). **(A)** Trial sequence. The frequencies of the standard pair were 30 and 300 Hz, while that of the comparison pair was 300 Hz. The presented frequency order of the standard pair (30 Hz first or 300 Hz first) was randomized. The presentation order of the standard and comparison pairs was randomized. **(B)** Averaged PSE of intervals between the standard pair. The left bar represents trials in which the standard pair was composed of a 300-Hz vibration followed by 30-Hz vibration; the right bar represents those in which it was composed of a 30-Hz vibration followed by 300-Hz vibration. The error bars represent 95% confidence intervals.

The final experiment was conducted to examine the perceived interval between paired vibrations with a different frequency range compared to those with the same frequency. In this experiment, the standard pair was a combination of 30- and 300-Hz vibrations, while the comparison pair consisted of 300-Hz vibrations (**Figure 3A**). The results showed that the apparent interval of the vibrations with the different frequency range was substantially overestimated (mean 179 ms, CI [160, 194] for 300 Hz first pair; mean 182 ms, CI [156, 216] for 30 Hz first pair) (**Figure 3B**). In addition, we found no significant difference due to the presented frequency order of the standard pair, 30 Hz first or 300 Hz first (the mean of the PSE difference according to the presented frequency order was 3.14 ms, CI [-18, 28]).

## Discussion

Tactile behavior studies have investigated the temporal perception of nearly simultaneous tactile stimuli using simultaneity or temporal order judgments and revealed that these judgments change depending on the stimulus attributes, such as the number and length of the stimuli and where on the body they are presented (Freeman and Johnson, 1982; Geffen et al., 2000; Yuan et al., 2006; Kuroki et al., 2010). However, few studies have investigated how these attributes effect the temporal perception of the sub-second time range. We examined whether the perceived temporal interval of vibration stimuli presented on the finger is altered simply by changing stimulus features. We found that the interval between a pair of vibrations presented on different fingers is overestimated relative to that between two vibrations presented on the same finger and that the interval between a weak pair is underestimated relative to a stronger pair. In addition, we found that the interval between a pair of vibrations with a different frequency/channel is overestimated relative to a pair of the same vibrations with the same frequency/channel. These results are consistent with our hypothesis that the interval between a bound pair of vibrations, in which the position is the same, the intensity is low, or the frequency is in the same range, is relatively underestimated compared to that between an unbound pair.

The stimulus location effect, or somatotopic distance effect, on tactile temporal tasks has been reported for simultaneity judgments (Clark and Geffen, 1990; Kuroki et al., 2010), temporal order judgments (Craig and Baihua, 1990; Kuroki et al., 2010), motion perception (Sherrick and Rogers, 1966; Sherrick, 1968; Kirman, 1974; Kuroki et al., 2010), and second-order time scale interval estimation (Kuroki et al., 2010). In this study, we clarified that the stimulus location indeed has a major effect on the tactile interval estimation of the sub-second range. As expected, we found that the estimated interval for stimuli on different fingers with 150-ms SOA significantly increased by around 40 ms from that for stimuli on the same finger. The present finding is consistent with the result of our previous study that examined this location effect on subjective interval estimation of 1 s (Kuroki et al., 2010). Though the difference did not reach a significant level at that time, the estimated interval of 1 s was overestimated by around 50 ms with different fingers, compared to that with the same finger. Since a misperception of time could also be induced by a misperception of localization, one may argue that our result could be explained by the kappa effect (Cohen et al., 1953; Henry et al., 2009), a well-known spatio-temporal illusion. With a sequence of more than three stimuli, participants tend to overestimate the apparent temporal interval between two of them when the spatial distance between them is longer than the other(s). This temporal mislocalization of the middle stimuli is due to its spatial relationship with the first and last stimuli. If the inter-stimulus distance has an effect on short-interval estimation only in the context of the kappa effect, the effect would vanish when the number of vibration stimuli is less than three. To verify this, we performed the same inter-stimulus distance effect experiment using auditory tones as comparison stimuli, which was almost the same procedure used in our previous research (Kuroki et al., 2010). Participants compared an interval between a pair of vibrations (standard, fixed at 150 ms) and that between a pair of tones (comparison, varied 75 to 300 ms), with a 30- or 300-Hz vibration pair presented on different (next) fingers or one finger. The mean perceived interval (PSE) of the 30-Hz vibration pair on two fingers was equivalent to 182 ms of the auditory pair, which was longer than that on one finger, which was 164 ms (CI of the PSE difference according to the presented fingers was [6.4, 31]). The PSE shift was also observed for the 300-Hz pair (185 ms vs. 155 ms, CI [20, 41]). Thus, the inter-stimulus distance seems to have an effect on tactile short-interval estimation outside the context of the kappa effect. Note that our finding is consistent with a previous study that suggested 500 ms as a critical time window for contextual effects for vision, audition, and also for touch (Burr et al., 2013). Since our first pair and second pair were separated by 1 s, we might not see the kappa effect. Still, we cannot exclude the possible contribution of spatial mislocalization to our results since we did not directly measure perceived locations. Whether the kappa effect occurred or not with our experimental conditions might be an interesting future investigation.

The intensity effect on duration perception has been reported in visual and auditory studies (Avant et al., 1975; Jaśkowski and Verleger, 2000; Terao et al., 2008; Boenke et al., 2009). Although whether the underlying mechanism is shared among modalities or not remains unknown, the present finding is consistent with the previously proposed hypothesis (Terao et al., 2008) that weak transient stimuli fail to tap the successiveness detector and brief interval judgments would be biased toward simultaneity. On the other hand, it has been suggested in tactile/visual studies that there is a deep relationship between perceived brief time and body movement (Yarrow et al., 2001; Park et al., 2003; Yarrow and Rothwell, 2003; Hagura et al., 2012; Tomassini et al., 2014). Since it is widely known that body movement also induces a reduction in tactile sensitivity, i.e., tactile sensory suppression (Milne et al., 1988; Williams et al., 1998), a possible relationship between sensory suppression and time compression has been suggested (Juravle, 2015). Although the current study did not explicitly address motion-induced sensory suppression, our results show that reducing stimulus intensity could cause apparent underestimation of inter-vibration intervals even without hand movement, which is consistent with the idea of this possible relationship. However, this is an issue awaiting further investigation. Tomassini et al. (2014), who clearly showed sub-second tactile time compression by hand movement, did an intensity control experiment. In their control experiment, the lowest intensity stimuli detectable during movement were presented to a static hand and an underestimation of inter-stimulus intervals was not observed. This apparent discrepancy in the results between their control experiment and our experiment 2 might be explained by the difference in the modulation ratio of the stimulus intensity. As weak stimuli, we used stimuli whose amplitude was halved, while they used almost 72% of the driving voltage to produce weak stimuli whose intensity was matched to the perceived intensity during body movement. In addition, there were differences in the frequency range of the stimuli. Some studies have suggested that high-frequency input suppresses responses of SI neurons to low-frequency inputs (Whitsel et al., 2001, 2003; Tommerdahl et al., 2005). We used band-limited low/high-frequency stimuli, while they used impulse stimuli that included a wide band of frequencies. This may also account for the difference. Still, both our study and Tomassini et al. (2014) did not fully control for the effect of sensory suppression, since neither tested an intensity-controlled condition under the influence of hand movement (Juravle, 2015). This is also an interesting direction for future study.

The frequency effect on interval judgment tasks has been examined in auditory studies. One study (Divenyi and Danner, 1977) used a procedure similar to ours, and the results are roughly consistent with the present ones. They reported that the frequency of short tones had no influence on the discriminability of an interval defined by two tones with the same frequency, which is in agreement with our not seeing any difference between the 30-Hz pair (dominantly RA channel) and 300-Hz pair (dominantly PC channel) in experiments 1 and 2. They reported impairment of temporal discrimination performance with tones different in frequency, while we found a PSE difference between the same and different frequency/channel pairs in experiment 3. In particular, we found that the interval between a pair that combined low-frequency “flutter” and high-frequency “vibration” is perceived to be longer than a pair with the same frequency/channel. It is conceivable, but unlikely, that the perceived timings of the onset of 30- and 300-Hz vibrations with the same duration are largely different. If, for example, the perceived onset “time marker” of 30 Hz is much earlier than that of 300 Hz, the perceived interval between the 30-Hz followed by 300-Hz pair should be sufficiently longer than that between 300-Hz followed by 30-Hz pair. We did not see such a trend in our results. It is possible that the time marker for the signals in the different frequency range might be different, but, even if so, it seems this would have had a minor effect in our tasks. Our results indicate a dominant effect on interval perception from the grouping/binding of multiple stimuli rather than on that from the difference in the latency of each stimulus.

The temporal illusion in this study presumably engaged the attention mechanism. Many studies have found that stimuli grabbing more transient attention or less predictable events are relatively overestimated, while those that engage less attention or predictable events are relatively underestimated (Rose and Summers, 1995; Mattes and Ulrich, 1998; Tse et al., 2004; Ono and Kawahara, 2007; Pariyadath and Eagleman, 2007; Yeshurun and Marom, 2008; Cicchini and Morrone, 2009). In addition, it is known that the perceived duration increases as a function of the complexity of stimuli (Schiffman and Bobko, 1974; Avant et al., 1975; Thomas and Weaver, 1975), where involvement of the attention mechanism can be considered. Since attention is allocated automatically to the abrupt onset of a new stimulus (Nakayama and Mackeben, 1989; Remington et al., 1992), our target pair in experiment 1, which was presented on different fingers, might be more salient. Our target pair in experiment 2, which was presented with half the amplitude, might be less salient compared with the control pair. Our target pair in experiment 3, which consisted of vibrations with different frequencies, might be more salient and less grouped. Our results are consistent with this notion: the interval between new/ungrouped stimuli are perceived to be longer than that between grouped stimuli. We also found a common binding effect when presented stimuli were 30 and 300 Hz for experiments 1 and 2, which is also consistent with the possibility that the illusion we found is mediated by higher-order processes, including the attention process. In addition, our results might parallel findings on gestalt illusions (Treisman, 1988; Duncan and Humphreys, 1989).

Although the main focus of this study was to assess behavioral changes associated with tactile binding/separation, there is a possible link between our results and intracortical interaction of neural activity. When two stimuli are presented within a brief interval, the first stimulus inhibits the amplitude level of somatosensory evoked activation for the second stimulus, and the effectiveness of this inhibition depends on the stimulus location, intensity, and frequency (Bystrzycka et al., 1977; Hsieh et al., 1995; David-Jürgens and Dinse, 2010). Accordingly, it can be speculated that strong intracortical inhibition occurred with a pair of stimuli close-in-space/within-channel in our experiment and that the perceived strength of the second stimulus might be weaker in these conditions, leading to their binding. This hypothesis still awaits further empirical validation.

In summary, we examined whether stimulus saliency can change perceived brief time, presented as an interval between vibrations, and found the following temporal illusions: separating the stimulus location caused apparent overestimation of the temporal interval of the two vibrations; reducing the stimulus intensity caused apparent underestimation; and separating the stimulus frequency/channel of the two vibrations caused apparent overestimation. These results suggest that two tactile events with low-saliency tend to be bound/grouped, which results in apparent compression of the perceived interval between events. Misperceptions of time are often ascribed to time differences in neural transmission or cortical processing, but can sometimes be ascribed to errors in encoding temporal relationships (Nishida and Johnston, 2002). Though further investigations are necessary, our results indicate that an underestimation of brief intervals in touch might also be caused by binding errors.

## Author Contributions

SK and JW contributed to conception and design of the experiments. SK collected and analyzed the data. SK drafted the paper, and TY and JW provided critical revisions.

## Conflict of Interest Statement

The authors SK, TY, and JW are employees of NTT Communication Science Laboratories, which is a basic-science research section of Nippon Telegraph and Telephone Corporation. There are no patents, products in development or marketed products to declare.
